# Hyperhomocysteinemia induced by excessive methionine intake promotes rupture of cerebral aneurysms in ovariectomized rats

**DOI:** 10.1186/s12974-016-0634-3

**Published:** 2016-06-27

**Authors:** Masaaki Korai, Keiko T. Kitazato, Yoshiteru Tada, Takeshi Miyamoto, Kenji Shimada, Nobuhisa Matsushita, Yasuhisa Kanematsu, Junichiro Satomi, Tomoki Hashimoto, Shinji Nagahiro

**Affiliations:** Department of Neurosurgery, Institute of Biomedical Sciences, Tokushima University Graduate School, 3-18-15 Kuramoto-cho, Tokushima, 770-8503 Japan; Department of Anesthesia and Perioperative Care, University of California, San Francisco, CA USA

**Keywords:** Hyperhomocysteinemia, Methionine, Cerebral aneurysm rupture, Anterior communicating artery, Macrophage, Matrix metalloproteinase-9, Tissue inhibitor of metalloproteinase-2, Folic acid

## Abstract

**Background:**

Hyperhomocysteinemia (HHcy) is associated with inflammation and a rise in the expression of matrix metalloproteinase-9 (MMP-9) in the vascular wall. However, the role of HHcy in the growth and rupture of cerebral aneurysms remains unclear.

**Methods:**

Thirteen-week-old female Sprague-Dawley rats were subject to bilateral ovariectomy and ligation of the right common carotid artery and fed an 8 % high-salt diet to induce cerebral aneurysms. Two weeks later, they underwent ligation of the bilateral posterior renal arteries. They were divided into two groups and methionine (MET) was or was not added to their drinking water. In another set of experiments, the role of folic acid (FA) against cerebral aneurysms was assessed.

**Results:**

During a 12-week observation period, subarachnoid hemorrhage due to aneurysm rupture was observed at the anterior communicating artery (AcomA) or the posterior half of the circle of Willis. HHcy induced by excessive MET intake significantly increased the incidence of ruptured aneurysms at 6–8 weeks. At the AcomA of rats treated with MET, we observed the promotion of aneurysmal growth and infiltration by M1 macrophages. Furthermore, the mRNA level of MMP-9, the ratio of MMP-9 to the tissue inhibitor of metalloproteinase-2, and the level of interleukin-6 were higher in these rats. Treatment with FA abolished the effect of MET, suggesting that the inflammatory response and vascular degradation at the AcomA is attributable to HHcy due to excessive MET intake.

**Conclusions:**

We first demonstrate that in hypertensive ovariectomized rats, HHcy induced by excessive MET intake may be associated with the propensity of the aneurysm wall to rupture.

**Electronic supplementary material:**

The online version of this article (doi:10.1186/s12974-016-0634-3) contains supplementary material, which is available to authorized users.

## Background

Subarachnoid hemorrhage (SAH) due to the rupture of cerebral aneurysms is a catastrophic event; its mortality rate is high despite advances in surgical techniques and perioperative management. Surgical clipping or endovascular coiling can be performed to prevent aneurysmal rupture. However, the mortality and morbidity rate after the clipping or coiling of unruptured aneurysms is not negligible [1]. A better understanding of the pathogenesis underlying the growth and rupture of cerebral aneurysms is needed to establish new therapeutic strategies.

Homocysteine (Hcy) is a non-protein-forming, sulphur-containing, non-essential amino acid that functions as a key intermediate in the methionine (MET) metabolism [2]. Hcy connects the MET- with the folate cycle. Several B vitamins play a role in the Hcy metabolism and an imbalance in the formation and removal of Hcy elicits changes in the plasma Hcy level. Some clinical studies suggested hyperhomocysteinemia (HHcy) as a strong independent factor in coronary disease, stroke, and abdominal aortic aneurysms [3–5]. Halazun et al. [6] reported that inflammation and matrix metalloproteinase (MMP)-2 and MMP-9 induced by HHcy are key factors in the growth of abdominal aortic aneurysms. Xu et al. [7] reported that in rats, HHcy induced by a MET-containing diet accelerated the formation of cerebral aneurysms, and Semmler et al. [8] suggested that polymorphisms affecting the Hcy metabolism were associated with the formation of cerebral aneurysms. However, the role of HHcy in the cerebral vascular structure and in the growth and rupture of cerebral aneurysms remains poorly understood.

Based on epidemiologic evidence of a high incidence of cerebral aneurysms in postmenopausal women, we established a cerebral aneurysm model in female rats subjected to hemodynamic stress, estrogen deficiency, and hypertension [9, 10]. We demonstrated that estrogen deficiency was associated with endothelial damage and that hypertension promoted the progression of cerebral aneurysms [11]. Activation of the local renin-angiotensin system (RAS), oxidative stress, inflammation, and sodium retention were associated with the formation of cerebral aneurysms [12–14]. Despite the high incidence of aneurysm formation at the anterior cerebral artery-olfactory artery (ACA-OA) bifurcation of rats, none of these aneurysms ruptured [9–15]. However, some aneurysms developed not only at the anterior communicating artery (AcomA) but also at the posterior half of the circle of Willis (PW), and they ruptured [15]. The PW includes the posterior communicating and the posterior cerebral artery (PcomA, PCA).

In humans, the hazard ratio of rupture was significantly higher for aneurysms at the AcomA and the PcomA than for middle cerebral artery (MCA) aneurysms [16]. A better understanding of the mechanisms underlying their growth and rupture is of clinical importance. We examined the role of HHcy in the growth and rupture of cerebral aneurysms at the AcomA and PW.

Here, we show that HHcy induced by excessive MET intake promoted the rupture of rat cerebral aneurysms at the AcomA and PW and the growth of aneurysms at the AcomA and that these events were associated with an increase in pro-inflammatory-and vascular degradation molecules.

## Methods

All procedures were performed with the rats under 2–4 % isoflurane inhalation anesthesia.

### Experimental animals

For aneurysm induction, 13-week-old female Sprague-Dawley rats were subjected to right common carotid artery (CCA) ligation to induce hemodynamic stress at the left ACA-OA bifurcation and the AcomA. They also underwent bilateral ovariectomy immediately after right CCA ligation. Starting on the next day, they were fed a high-salt diet (8 % sodium chloride). Two weeks later, they were subjected to ligation of the bilateral posterior renal arteries to induce hypertension. The time point of aneurysm induction was defined as the time of bilateral posterior renal artery ligation.

*Experiment 1*: To assess the incidence of SAH and the morphological aneurysmal wall changes, we prepared two groups. One group (MET, *n* = 25) did, and the other (control, *n* = 19) did not receive 4 mg/ml MET (Wako, Osaka, Japan) in their drinking water. At 12 weeks after aneurysm induction, we prepared vascular corrosion casts of all asymptomatic rats. In a separate experiment, another set of rats (MET, *n* = 6; control, *n* = 6) was used to analyze the mRNA level by quantitative real-time polymerase chain reaction (qRT-PCR) assay and the results were compared with sham-operated rats (sham, *n* = 6) fed with a standard diet including 0.3 % sodium chloride.

*Experiment 2:* To test whether folic acid (FA) prevents the growth and rupture of cerebral aneurysms, another set of 19 MET rats was fed FA (Sigma-Aldrich, Tokyo, Japan) daily (4 mg/kg; MET + FA rats) to attenuate the conversion from MET to Hcy. They were compared with 35 MET-only rats. The administration of FA and MET was started at the same time. We also prepared six additional rats in each group for qRT-PCR assay. The MET and FA doses were based on findings reported in earlier studies [7, 17, 18].

Rats that died within 4 weeks after aneurysm induction were excluded. As 2 to 8 weeks are needed for plasma Hcy to return to the normal level in rats given a diet containing FA [18], we began evaluating its effects on aneurysmal rupture 4 weeks after the start of FA administration. The observation period was 12 weeks. To detect aneurysmal rupture, two blinded observers performed daily neurological examinations and recorded neurological signs, i.e., motor deficits, seizures, absence of spontaneous activity, and death. The brains of all symptomatic rats harbored cerebral aneurysms and manifested SAH. None of the asymptomatic rats that survived for 12 weeks suffered SAH. We defined the incidence of ruptured aneurysms as the ratio of rats with ruptured aneurysms to the total number of rats. Ruptured aneurysms were observed under a stereomicroscope after removing the SAH thrombus.

### Blood pressure and plasma Hcy measurements

For blood pressure readings, once every 4 weeks, unanesthetized rats were placed on a 37 °C hot plate (NISSIN, Tokyo, Japan) and covered with a black blanket. After acclimatization, one blinded examiner recorded their average blood pressure based on three measurements obtained with the tail-cuff method (Softron, Tokyo, Japan). Blood was withdrawn 12 weeks after the last procedure; plasma samples were stored at −80 °C until use. In humans, plasma Hcy levels exceeding 15 nmol are recorded as HHcy [19].

### Preparation of vascular corrosion casts

Vascular corrosion casts were prepared as previously described [20]. Rats were transcardially perfused with heparinized phosphate-buffered saline (PBS) followed by the injection of Batson’s No. 17 plastic (Polyscience Inc., Warrington, PA, USA). The left ACA-OA bifurcation and the AcomA on the casts were inspected at 3 kV under a scanning electron microscope (VE8800, Keyence, Osaka, Japan). Morphological changes at the AcomA were evaluated by three blinded observers and classified into four stages where stage 0 = no arterial dilation or irregular shapes, stage 1 = slight protrusion (diameter of the aneurysmal protrusion < half of the diameter of the parent artery), stage 2 = moderate protrusion (half the diameter of the parent artery < diameter of the protrusion < diameter of the parent artery), and stage 3 = a well-developed saccular aneurysm (diameter of the aneurysm > diameter of the parent artery).

### RNA isolation and qRT-PCR assay

The mRNA levels in the vascular wall from sham, control, and MET rats (*n* = 6 in each group) were determined by qRT-PCR assay. The mRNA levels in another set of MET- and MET + FA rats (*n* = 6 in each group) were also compared. The rats were euthanized 12 weeks after aneurysm induction. The AcomA and the left ACA-OA bifurcation were isolated and total RNA was extracted with the EZ1 RNA Universal Tissue Kit (QIAGEN, Tokyo, Japan) and placed in a MagNA lyser (Roche, Tokyo, Japan). For reverse transcription of total RNA to cDNA, we used the transcriptor first-strand cDNA synthesis kit (Roche). qRT-PCR assay of each sample was in a LightCycler 2.0 instrument (Roche Diagnostics, Tokyo, Japan). LightCycler FastStart DNA master and SYBR green I (Roche) were used for MMP-9, tissue inhibitor of metalloproteinase (TIMP)1, TIMP2, NOX4, Rac1, angiotensin-converting enzyme (ACE), tumor necrosis factor (TNF)α, interleukin (IL)-6, and glyceraldehyde-3 phosphate dehydrogenase (GAPDH) assays. The primers were

- For rat MMP-9: forward primer (F), 5′-ACA ACG TCT TTC ACT ACC AA-3′, reverse primer (R), 5′-CAA AAG AGG AGC CTT AGT TC-3′
- for TIMP1: F, 5′-TCC TGG TTC CCT GGC ATA AT-3′, R, 5′-GGC AAA GTG ATC GCT CTG GT-3′
- for TIMP2: F, 5′-CCC TCT GTG ACT TTA TTG TGC-3′, R, 5′-TGA TGC TCT TCT CTG TGA CC-3′
- for NOX4: F, 5′-AGA CAT CCA ATC ATT CCA GTG GTT TGC AGA C-3′, R, 5′-TGC TCT ATG TGC TGC ATA ACA AGT TTT GGC AA-3′
- for Rac1: F, 5′-CTG TCT TGA GTC CTC GCT GTG TGA GTG CTG-3′, R, 5′-CAG CAG GCA TTT TCT CTT CC-3′
- for TNFα, F, 5′-CCC AAC AAG GAG GAG AAG T-3′, R, 5′-CGC TTG GTG GTT TGC TAC-3′
- for IL-6, F, 5′-TCT CAG GGA GAT CTT GGA AAT G-3′, R, 5′-TAG AAA CGG AAC TCC AGA AGA C-3′
- for GAPDH, F, 5′-TAC ACT GAG GAC CAG GTT G-3′, R, 5′-CCC TGT TGC TGT AGC CAT A-3′.

Primers and probe sets for ACE were from Roche and used according to the manufacturer’s directions. The amplified product was separated on 1.5 % agarose gels containing EtBr solution (Wako, Osaka, Japan) and visualized on an ultraviolet transilluminator. The results were quantified after normalization to the expression of GAPDH mRNA. The PCR conditions were 95 °C for 10 min followed by 40 cycles at 95 °C for 10 s, 60 °C for 10 s, and 72 °C for 8 s. We subjected samples from each group to two independent qRT-PCR assays. GAPDH was the internal control.

### Immunofluorescent studies

In a separate set of experiments, 3 MET and 3 control rats were prepared for immunofluorescence studies. After perfusion with 4 % paraformaldehyde, we inspected the vessels in the circle of Willis under a dissecting microscope. The AcomA was carefully dissected and immersed in 4 % paraformaldehyde. The arteries were rinsed with PBS, embedded in OCT compound (Tissue-Tek, Inc.), and 6-μm-thick serial sections were cut with a cryotome (CM 1850; Leica). After 30-min serum-free protein blockade (Dako, Carpinteria, CA, USA), primary antibodies diluted with Canget signal immunostain (Toyobo, Osaka, Japan) were added for 1-h incubation at room temperature (RT) or overnight at 4 °C. Primary antibodies against MMP-9, CD163 (Abcam, CA, USA), CD11b (BioLegend, CA, USA), and CD68 (Abbiotec, CA, USA) were applied. Sections were then incubated for 1 h at RT with the fluorescein-conjugated secondary antibodies Alexa Fluor 488 or 594 (Molecular Probes, CA, USA) in Canget signal immunostain, mounted with Vectashield (Vector Laboratories, CA, USA), and examined under a fluorescence microscope (Olympus IX71, Tokyo, Japan).

### Statistical analysis

Sequentially obtained data (mean ± SD) were analyzed with Student’s *t* test for 2-group comparisons and analysis of variance (ANOVA) followed by Scheffe’s test for multiple comparisons. The incidence of ruptured aneurysms and of cerebral aneurysmal changes was analyzed with Fisher’s exact probability test. Statistical analyses were performed with statistical software (StatView 5). Differences were considered statistically significant at *p* < 0.05.

## Results

### Excessive MET intake promotes the rupture of rat cerebral aneurysms

**Fig. 1:**
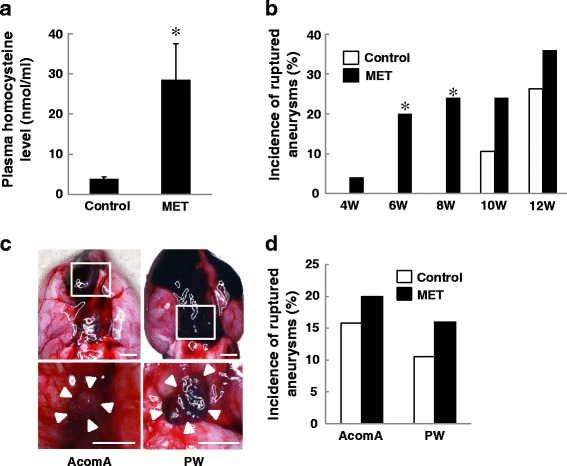
Excessive methionine (MET) intake increased the incidence of cerebral aneurysmal rupture. **a** The plasma homocysteine (Hcy) level in MET-treated rats was significantly higher than in the controls. Plasma Hcy was measured by radioimmunoassay (each group, *n* = 5). *p < 0.01 vs control rats by Student’s *t* test (mean ± SD). **b** The total rupture rate was significantly higher in MET-treated rats (*n* = 25) than in the controls (*n* = 19) at 6 and 8 weeks after aneurysm induction. *p < 0.05 vs control rats by Fisher’s exact probability test. **c** Representative photographs of subarachnoid hemorrhage (SAH) due to the rupture of aneurysms at the anterior communicating artery (AcomA) (*left*) and the posterior half of the circle of Willis (PW) (*right*). Aneurysm formations were found after the removal of the blood clot from SAH (*lower photographs*). The location of aneurysms is indicated by *rectangles. White arrows* indicate aneurysms. Bars = 2.5 mm. **d** The incidence of rupture of AcomA and PW aneurysms in MET-treated and control rats

MET forms Hcy via numerous adenosylmethionine-dependent methyl transfer reactions. The plasma Hcy level was significantly higher in MET-than control rats (*p* < 0.01, Fig. 1a), confirming HHcy in the MET rats. The average MET intake in the course of 12 weeks was 772 ± 118 mg/kg/day. In MET-and control rats, we observed hypertension at 4 weeks after aneurysm induction (Additional file 1: Figure S1A). The blood pressure and body weight were not affected by MET (Additional file 1: Figure S1A, S1B). During the 12-week post-induction observation period, the incidence of aneurysmal rupture was significantly higher at 6 and 8 weeks; the total incidence tended to be higher in MET than control rats at 10 and 12 weeks (Fig. 1b). Aneurysmal rupture sites were observed at the AcomA and the PW (Fig. 1c, d). We observed no ruptures at the ACA-OA bifurcation. These results suggest that excessive MET intake promoted aneurysmal rupture at the AcomA and PW.

### Excessive MET intake may increase the vulnerability of the AcomA wall

**Fig. 2:**
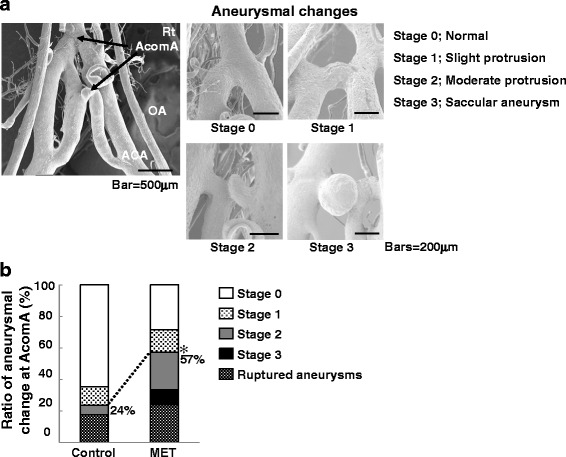
Evaluation of wall changes at the AcomA on vascular corrosion casts. **a** Changes were divided into four stages where 0 = no arterial dilation or irregular shapes, stage 1 = slight protrusion (diameter of the aneurysmal protrusion < half of the diameter of the parent artery), stage 2 = moderate protrusion (half the diameter of the parent artery < diameter of the protrusion < diameter of parent artery), and stage 3 = a well-developed saccular aneurysm (diameter of the aneurysm > diameter of the parent artery). **b** The incidence of stage 2 and stage 3 changes and of rupture at the AcomA was significantly higher in methionine (MET)-treated than control rats. MET-treated vs. control: 57 vs 24 %, *p < 0.05 by Fisher’s exact probability test

Since the incidence of rupture was almost equal at the AcomA and the PW, we examined the morphology at the AcomA to identify factors that render the vessel wall prone to rupture. We classified the aneurysmal changes observed on corrosion cast into four stages (Fig. 2a). There were significantly more stage 2 and 3 changes and more ruptured aneurysms in the MET-than the control rats (Fig. 2b), suggesting that excessive MET intake promotes the growth and rupture of AcomA aneurysms. There was no significant difference in the rate of aneurysm formation at the ACA-OA bifurcation between control-and MET rats (Additional file 2: Figure S2A). HHcy induced by overdosing with MET may exacerbate the site-specific vulnerability of the vessel wall and aneurysmal instability.

### Excessive MET intake is associated with increased M1 macrophage infiltration into the AcomA wall and with vascular degradation

**Fig. 3:**
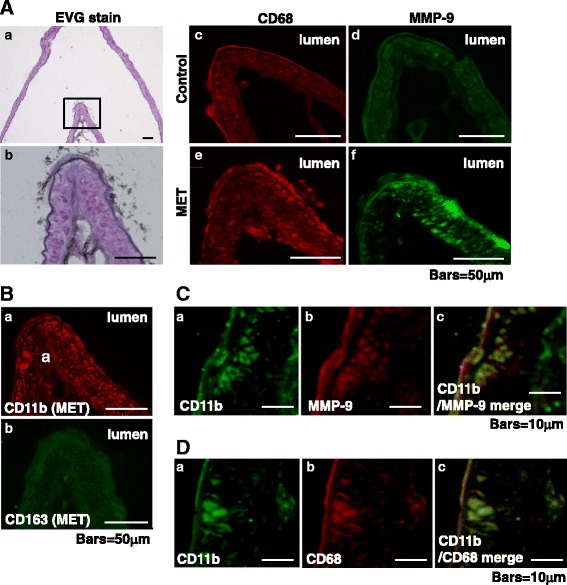
Evaluation of pathological changes at the AcomA wall by Elastica van Gieson and immunofluorescence staining. **a** EVG (*a*, *b*) and immunofluorescence staining for CD68 (*c*, *red*) and MMP-9 (*d*, *green*) in control rats and for CD68 (*e*, *red*) and MMP-9 (*f*, *green*) in methionine (MET)-treated rats. **b** Immunofluorescence staining for CD11b (*a*, *red*), CD163 (*b*, *green*) in MET-treated rats. **c** Immunofluorescence staining for CD11b (*a*, *green*), MMP-9 (*b*, *red*), and merged stains (*c*) in MET-treated rats. **d** Immunofluorescence staining for CD11b (*a*, *green*), CD68 (*b*, *red*), and merged stains (*c*) in MET-treated rats

Immunohistochemically, CD68-positive cells are peripheral macrophages and CD11b and CD163 are expressed in differentiated M1 and M2 macrophages, respectively. M1 and M2 macrophages are associated with pro-inflammatory- and anti-inflammatory responses, respectively. CD68- and CD11b- but not CD163-positive cells were increased in MET rats and they were co-localized (Fig. 3a, b, d). MMP-9-positive cells were also increased in MET-(Fig. 3a) but not in control rats. They co-localized with CD11b-positive cells (Fig. 3c).

**Fig. 4:**
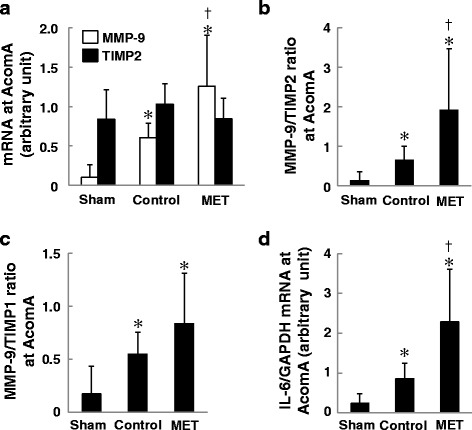
The mRNA level at the AcomA (*n* = 6 for each group). **a** The mRNA expression of MMP-9 was significantly higher in methionine (MET)-treated than in control and sham-operated rats. **b** The MMP-9 to TIMP2 ratio was significantly higher in MET-treated than control and sham rats. **c** The MMP-9 to TIMP1 ratio was not significantly different between MET-treated and control rats. **d**. The mRNA expression of IL-6 was significantly higher in MET-treated than in control and sham rats. The mRNA levels were normalized by the GAPDH mRNA level. *p < 0.05 vs sham- and ^†^ *p* < 0.05 vs control rats by ANOVA followed by Scheffe’s test (mean ± SD)

The mRNA level of MMP-9, the ratio of MMP-9 to TIMP2, and the IL-6 level were significantly higher in MET-than control rats (Fig. 4a, b, d). The MMP-9 to TIMP1 ratio at the AcomA (Fig. 4c) and the ratio of MMP-9 to TIMP2 at the ACA-OA bifurcation (Additional file 2: Figure S2B) were not significantly different in MET and control rats. The increase in MMP-9, the disequilibrium in the MMP-9 to TIMP2 ratio, and the infiltration of M1 macrophages may be associated with the vulnerability of the AcomA wall.

**Fig. 5:**
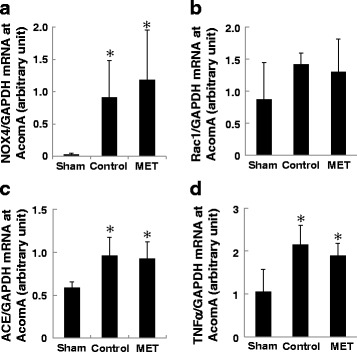
qRT-PCR assay of samples from the AcomA (*n* = 6 for each group). Methionine (MET) intake did not affect the mRNA level of NOX4 (**a**), Rac1 (**b**), ACE (**c**), and TNFα (**d**). The mRNA levels were normalized by the GAPDH mRNA level. *p < 0.05 vs sham rats by ANOVA followed by Scheffe’s test (mean ± SD)

The mRNA level of NOX4 (Fig. 5a), ACE (Fig. 5c), and TNFα (Fig. 5d) was higher in the AcomA wall of control-and MET than of sham rats. There was no significant difference between control and MET rats. The mRNA level of Rac1 (Fig. 5b) in control- and MET rats was similar to the sham rats. These observations suggest that the vascular wall in both groups was exposed to a similar degree of oxidative stress and RAS activation and that these factors may not be directly associated with aneurysmal growth or rupture.

### The inhibition of conversion from MET to Hcy attenuates the development of AcomA aneurysms by inhibiting vascular degradation

**Fig. 6:**
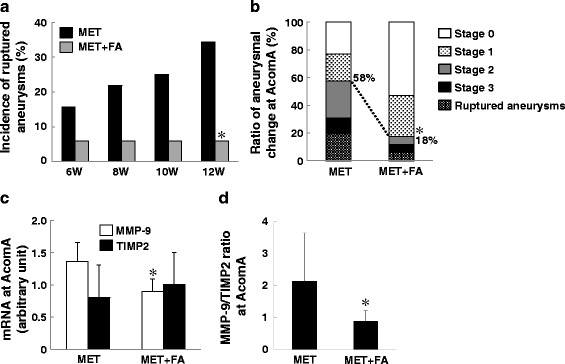
Folic acid (FA) attenuated the development of cerebral aneurysms. **a** Methionine (MET)-only (*n* = 32) and MET + FA-treated rats (*n* = 17). FA lowered the total rupture rate observed at 12 weeks after aneurysmal induction. *p < 0.05 vs MET-only rats by Fisher’s exact probability test. **b** The total rates of stage 2 and stage 3 aneurysmal wall changes and of rupture were significantly lower in MET + FA than in MET-only rats (18 vs 58 %, *p < 0.01 vs MET-only rats by Fisher’s exact probability test). **c**, **d** FA decreased the expression of MMP-9 and of the MMP-9 to TIMP2 ratio. *p < 0.05 vs MET-only rats by Student’s *t* test

Hcy is degraded by FA. We treated MET rats with FA (MET + FA) to confirm the relationship between HHcy and aneurysms at the AcomA. As expected, their plasma Hcy level was normalized by FA (Additional file 3: Figure S3A); it did not affect the systolic blood pressure (Additional file 3: Figure S3B). The rupture rate was significantly lower in MET + FA-than MET-only rats 12 weeks after aneurysm induction (Fig. 6a) and the incidence of stage 2 and 3 morphologic changes and of ruptured AcomA aneurysms was significantly lower in MET + FA than MET-only rats (Fig. 6b). The increase in the expression of MMP-9 and in the ratio of MMP-9 to TIMP2 at the AcomA seen in the presence of HHcy was abolished (Fig. 6c, d). These results suggest that the degradation of the AcomA wall is attributable to HHcy.

## Discussion

In this study, we first demonstrate that HHcy induced by excessive MET intake promotes the growth of AcomA-and the rupture of AcomA and PW aneurysms. In MET rats, we observed an increase in the infiltration of M1 macrophages, the expression of MMP-9, and the ratio of MMP-9 to TIMP2 at the AcomA. Treatment with FA reduced HHcy, attenuated the effects of MET, and prevented the growth and rupture of aneurysms. Our findings suggest that the vulnerability of the AcomA wall in MET rats is at least partly attributable to HHcy and that its suppression may help to prevent the growth and/or rupture of cerebral aneurysms.

Up to 40 % of patients diagnosed with premature coronary artery disease, peripheral vascular disease, or recurrent venous thrombosis manifested HHcy [21, 22]. However, it is unclear whether HHcy is a causative factor or a result of these diseases. In mice, the oral administration of FA or of a combination of B vitamins decreased HHcy and attenuated atherogenesis [23]. These observations support our present findings that HHcy promotes the growth and rupture of cerebral aneurysms.

In humans, efforts to lower HHcy by therapy with FA and vitamin B did not reduce the incidence of cardiovascular events [22, 24]. The daily combined intake of FA and vitamins B6 and B12 for 5 years failed to significantly reduce the rate of major vascular events in a high-risk population with vascular disease, possibly because it failed to reverse epigenetic changes induced by HHcy [24]. Saposnik et al. [25] found that while FA helped to lower Hcy and vitamins B6 and B12 reduced the overall risk for stroke, there was no effect on stroke severity and post-stroke disability. On the other hand, among adult hypertensive Chinese with no history of stroke or myocardial infarction, the combined use of enalapril, an angiotensin-converting enzyme inhibitor, and FA significantly reduced the risk for a first stroke, compared to patients treated with enalapril alone [26]. Epigenetic factors may contribute differently to cardiovascular and cerebrovascular diseases. Combination therapies aimed at improving the epigenetic machinery and lowering circulating Hcy levels may be more beneficial. Since the blood pressure in our MET + FA rats was not affected, we posit that HHcy contributed to the growth and rupture of their cerebral aneurysms in a blood pressure-independent manner.

Many macrophages were observed in the human luminal and abluminal layer of large unruptured and ruptured aneurysms [27, 28]. Elsewhere, we demonstrated that in our rat aneurysm model, the degradation of endothelial tight junctions facilitated macrophage infiltration into the vascular wall [29]. While pro-inflammatory M1 and anti-inflammatory M2 macrophages were present in equal proportion in unruptured aneurysms, the number of M1 macrophages was increased in ruptured aneurysms [30]. Yao et al. reported that IL-6 increased MMP-9 activity in human cerebral smooth muscle cells in vitro [31]. Tissue-infiltrating macrophages not only release pro-inflammatory cytokines that lead to the recruitment of additional inflammatory cells, but they also release MMPs that digest the extracellular matrix of the arterial wall, resulting in further damage via the up-regulation of other proteinases. Thus, the infiltration of macrophages and macrophage-derived MMPs may be closely associated with aneurysmal growth. The increase in CD68^+^/CD11b^+^ macrophages and the elevated mRNA level of IL-6 in the AcomA wall of MET rats may reflect this phenomenon.

On the other hand, others [32, 33] reported that the highest wall shear stress involved the AcomA rather than other cerebral arterial bifurcations and that it was highly oscillatory. Oscillatory wall shear stress increased the expression of MMP-9 in endothelial cells [34] and Sho et al. [35] reported that high flow and shear stress can induce endothelial and smooth muscle cells to express MMP-2 and MMP-9; they also documented a disproportional increase in TIMP2. As we did not directly assess the blood flow, we cannot comment on the relationship between hemodynamics and the propensity of AcomA aneurysms to rupture. Whether the imbalance between MMP-9 and TIMP2 at the AcomA is associated with complex hemodynamics remains to be determined. The expression of macrophage-derived MMP-9 is critical for vascular wall remodeling. It was significantly increased in the wall of cerebral aneurysms in rats exposed to HHcy [7] and the selective inhibition of MMP blocked aneurysmal progression [36]. Therapies targeting MMP-9 and TIMP2 may be promising candidates in efforts to prevent the growth and rupture of cerebral aneurysms.

Elsewhere [11–15], we reported that RAS, oxidative stress, and inflammation were strongly associated with the formation of cerebral aneurysms. In the current study, the expression of these molecules was higher in both our MET and control rats than in the sham rats. However, there was no significant difference between MET and control rats although the MET rats harbored a significantly higher grade of AcomA aneurysms than the controls, suggesting that these molecules may be associated with the formation of cerebral aneurysms but that they may not be directly associated with aneurysmal growth or rupture.

Our study has some limitations. As it was difficult to obtain samples just after rupture, we could not assess differences in the molecule levels between ruptured and unruptured AcomA aneurysms. We also did not determine whether the AcomA used for the analysis of the mRNA level harbored aneurysms. Although we document the relationship between the increase in degradation molecules and AcomA aneurysms with an unstable wall, we were unable to identify the detailed mechanisms underlying aneurysmal rupture. Since we excluded rats with ruptured aneurysms at the PW, there is sampling bias in assessment of aneurysmal changes at AcomA (Figs. 2b and 6b).

## Conclusions

We provide new evidence that HHcy elicited by an excessive MET intake is associated with the vulnerability of the vascular wall at the AcomA. Suppressing inflammation, vascular degradation, and macrophage infiltration may prevent aneurysmal rupture. Our findings may pave the way for the development of pharmacological therapies to address human cerebral aneurysms.

## Abbreviations

ACA-OA, anterior cerebral artery-olfactory artery; ACE, angiotensin-converting enzyme; AcomA, anterior communicating artery; CCA, common carotid artery; FA, folic acid; Hcy, homocysteine; HHcy, hyperhomocysteinemia; IL, interleukin; MMP, matrix metalloproteinase; PW, posterior half of the circle of Willis; qRT-PCR, quantitative real-time polymerase chain reaction; RAS, renin-angiotensin system; SAH, subarachnoid hemorrhage; TIMP, tissue inhibitor of metalloproteinase; TNF, tumor necrosis factor

## Additional files

Additional file 1: Systolic blood pressure and body weight. (PDF 78 kb) Additional file 2: Incidence of aneurysms and the MMP-9 to TIMP2 ratio at ACA-OA. (PDF 77 kb) Additional file 3: Plasma homocysteine level and systolic blood pressure. (PDF 35 kb)
