# Supplementary material for: Hyperhomocysteinemia induced by excessive methionine intake promotes rupture of cerebral aneurysms in ovariectomized rats
Source: J Neuroinflammation. 2016 Jun 27;13:165. doi: 10.1186/s12974-016-0634-3 (PMC4924228; doi:10.1186/s12974-016-0634-3)
Supplement: Additional file 1: — Systolic blood pressure and body weight. (PDF 78 kb) [file 12974_2016_634_MOESM1_ESM.pdf]

Figure S1

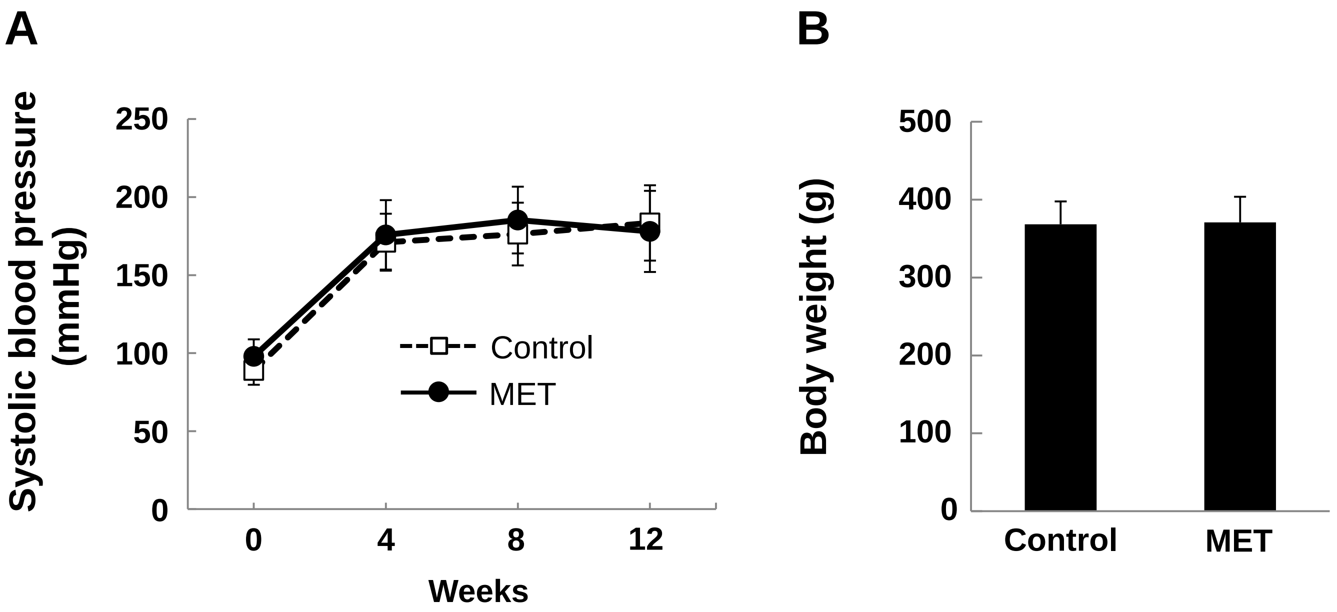

A. The systolic blood pressure was measured at 4-week intervals with the tail-cuff method. There was no difference between the two groups. Data are the mean ± SD.

B. The body weight 12 weeks after bilateral posterior renal artery ligation was recorded. Data are the mean ± SD for 10 rats in both groups.
