# Supplementary material for: Hyperhomocysteinemia induced by excessive methionine intake promotes rupture of cerebral aneurysms in ovariectomized rats
Source: J Neuroinflammation. 2016 Jun 27;13:165. doi: 10.1186/s12974-016-0634-3 (PMC4924228; doi:10.1186/s12974-016-0634-3)
Supplement: Additional file 2: — Incidence of aneurysms and the MMP-9 to TIMP2 ratio at ACA-OA. (PDF 77 kb) [file 12974_2016_634_MOESM2_ESM.pdf]

Figure S2

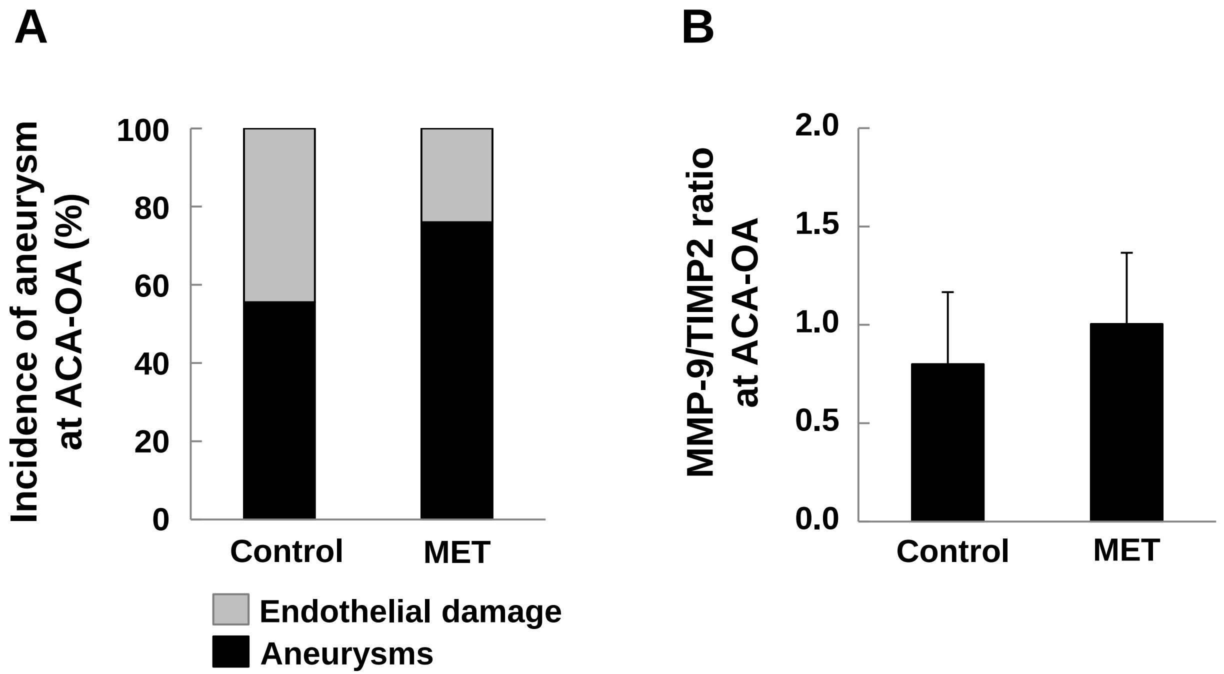

A. Incidence of aneurysms at the anterior cerebral artery-olfactory artery (ACA-OA) bifurcation in control- (n=14) and methionine (MET)- (n=16) treated rats. Rat brain vascular corrosion casts were inspected under a scanning electron microscope. Endothelial damage was recorded when the casts manifested irregularly-shaped cell imprints.

B. By qRT-PCR assay, the ratio of matrix metalloproteinase (MMP)-9 to tissue inhibitor of metalloproteinase (TIMP)2 at the ACA-OA bifurcation was not significantly different in the 2 groups.

Data analysis was with the Fisher exact test (**A**) and the Student *t*-test (both groups, n=6, mean ± SD)
