# Supplementary material for: Hyperhomocysteinemia induced by excessive methionine intake promotes rupture of cerebral aneurysms in ovariectomized rats
Source: J Neuroinflammation. 2016 Jun 27;13:165. doi: 10.1186/s12974-016-0634-3 (PMC4924228; doi:10.1186/s12974-016-0634-3)
Supplement: Additional file 3: — Plasma homocysteine level and systolic blood pressure. (PDF 35 kb) [file 12974_2016_634_MOESM3_ESM.pdf]

Figure S3

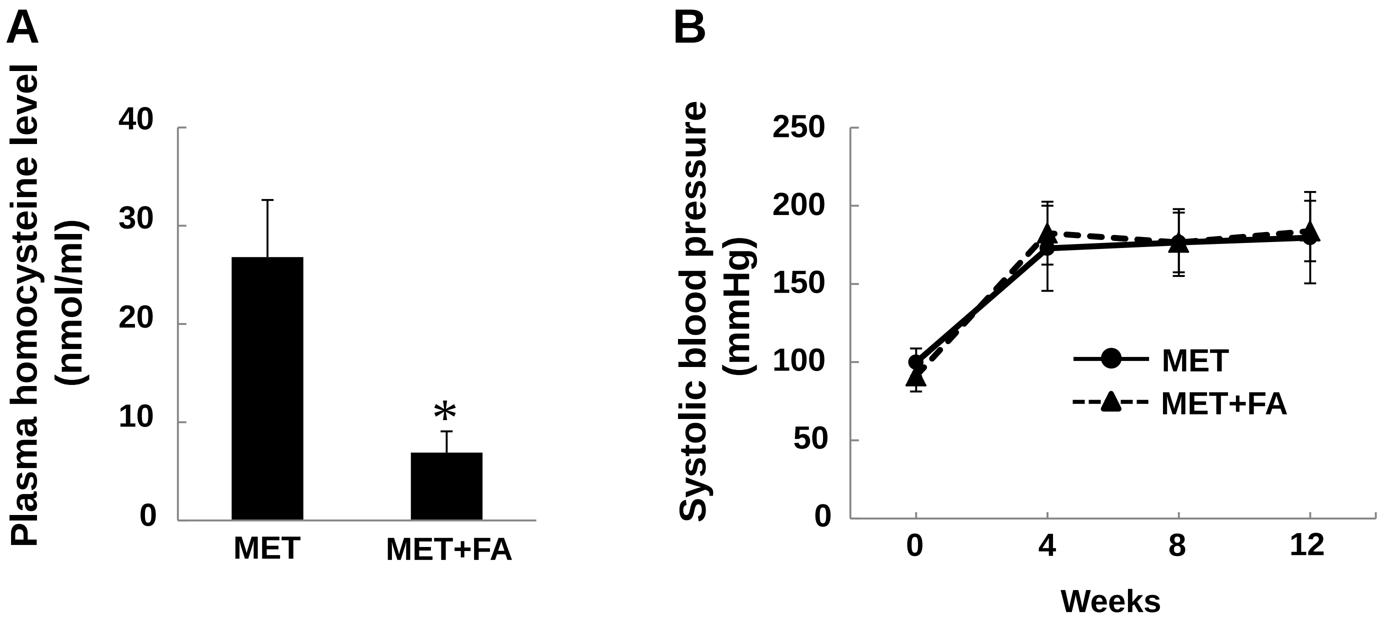

A. Plasma homocysteine level measured by radioimmunoassay.

*p<0.05 vs methionine (MET)-treated rats. Student's *t* test (each group, n=5, mean ± SD).

B. The systolic blood pressure was measured every 4 weeks using the tail-cuff method. Folic acid did not affect the systolic blood pressure. Data are the mean ± SD in both groups.

MET+FA: Rats treated with MET plus folic acid.
